# Feeling the Vibes: An Investigation Into Resident Antibiotic Prescribing Practices

**DOI:** 10.1093/ofid/ofag017

**Published:** 2026-01-23

**Authors:** David A Dickson, Jaime M Jordan, Tara Vijayan

**Affiliations:** Department of Internal Medicine, University of California, Los Angeles, California, USA; Department of Infectious Diseases and Geographic Medicine, Stanford University, Stanford, California, USA; Department of Emergency Medicine, University of California, Los Angeles, California, USA; Department of Emergency Medicine, Oregon Health & Science University, Portland, Oregon, USA; Division of Infectious Diseases, University of California, Los Angeles, California, USA

**Keywords:** antibiotic decision making, antimicrobial stewardship, internal medicine residents, medical education, navigating uncertainty

## Abstract

**Background:**

Efforts to improve inpatient antibiotic prescribing are limited by a lack of insight into the complicated decisions around antibiotic use. We aimed to explore antibiotic therapeutic decision making among internal medicine resident physicians.

**Methods:**

We performed a qualitative study with a constructivist paradigm employing semistructured in-person focus groups of internal medicine trainees at a teaching hospital system from December 2023 through January 2024. Two researchers independently performed a thematic analysis of focus group transcripts. We resolved discrepancies through in-depth discussion, negotiated consensus, and converged codes into overarching themes.

**Results:**

Twenty-five residents participated across 3 focus groups. Residents identified a general approach to prescribing empiric antibiotics, including triaging critical illness and identifying the presence of infection, the source of infection, the antibiotic that covers the likely pathogens, and relevant patient-specific factors. Empiric choice was modulated by 3 subthemes: institutional culture, antibiotic stewardship policies, and clinical resources. Major challenges in therapeutic decision making included navigating uncertainty, fear of clinical deterioration, difficulty determining appropriate antibiotic duration/spectrum, and the inconsistency of clinical reasoning by supervising attendings. Certain safety net strategies were used to mitigate this uncertainty. Residents felt that their confidence in antibiotic prescribing decisions improved over time through experience, especially on overnight rotations. Infectious diseases physicians and pharmacists provided education and a needed model approach for therapeutic reasoning and supported residents in increasing their risk tolerance.

**Conclusions:**

This study provides insights into resident decision making regarding antibiotic use, which may inform educational interventions to optimize antibiotic utilization and adherence to practice guidelines at teaching hospitals.

## Teaser

Despite articulating a systematic approach to antibiotic prescribing, resident physicians struggle with navigating uncertainty and fear of patients’ clinical deterioration. Understanding these barriers may allow tailored educational interventions in antibiotic stewardship and improve comfort with nuanced therapeutic decision making.

The Centers for Disease Control and Prevention estimates that more than half of antimicrobial prescribing events in hospitals are not consistent with recommended guidelines [1]. Given the ubiquity of antibiotics in the inpatient hospital setting, efforts at curbing the inappropriate use of broad-spectrum antimicrobials have been difficult to implement. Reviews on this topic note a clear need to better understand the antibiotic utilization of health care providers and identify effective interventions to improve practice and strategies for implementation locally and across diverse settings [2, 3]. A noted challenge to the success of these interventions is an incomplete understanding of the thought processes that influence antibiotic use and selection.

Recent studies on therapeutic decision making have identified critical steps of how specific antibiotics are selected [4, 5], and systematic reviews have identified a variety of factors influencing prescribing choices across domains, including those related to the prescriber, the patient, the medications, and other external factors [6, 7]. Of the studies on physicians, the majority of these reviews have focused mainly on attendings [4–7], with only a fraction including studies on physician trainees [8–10]. One recent observational study suggested that junior residents, when compared with senior residents and attendings, prescribe significantly more broad-spectrum antibiotics, including those with activity against methicillin-resistant *Staphylococcus aureus* and *Pseudomonas*, despite similar patient complexity [11]. Other studies suggest that residents do not readily tie the risks of such broad prescribing into increased patient morbidity and mortality [12], and even attending physicians perceive that broad-spectrum antibiotics are “low risk” [7]. Survey data show that residents acknowledge the importance of rising antibiotic resistance yet lack confidence in their own education on antibiotic prescribing [13, 14]. While there are known hierarchical and interprofessional challenges specific to trainees that likely contribute to these differences in antibiotic prescribing, these data suggest a significant opportunity for improvement [15].

A more robust understanding of clinical decision making surrounding antibiotic use in trainees is essential to identify targets for intervention during residency and improve overall antibiotic stewardship for teaching hospitals. We sought to explore internal medicine (IM) resident decision making surrounding antibiotic use.

## METHODS

### Participants, Recruitment, and Setting

We recruited postgraduate year 1-4 residents (eg, categorical, IM/pediatrics, and transitional year interns) and chief residents currently enrolled in the IM or combined IM/pediatrics training program at a large urban academic medical center in the western United States. We chose IM residents as they represent a large proportion of the prescribers in academic hospitals and are expected to have mastery in the management of the most common infectious diseases (ID) by the end of training. We included residents from all training years and utilized focus groups to better capture the multifaceted and collaborative nature of IM rounds. Focus groups also allowed for increased participant convenience and inclusion as compared with individual interviews, as they were performed during normal noon conference lecture slots during which trainees would typically be expected to attend. We chose antibiotics, as opposed to the broader term *antimicrobials*, for 2 reasons: first, residents would be expected to have significantly more personal experience prescribing antibiotics as compared with antivirals and antifungals; second, the term *antibiotics* is more commonly used, despite its imprecision [16]. Residents were allowed to participate in only 1 focus group. One week prior to each focus group, we emailed all residents currently on service at the hospital an invitation to participate, including brief details. We conducted in-person focus groups in a private room at the hospital. Attendance at the sessions was voluntary. Participants were allowed to leave at any time and have their responses/contributions removed from the study by request at any point. We did not provide any compensation for participation.

### Study Design

We performed a qualitative study employing thematic analysis with a constructivist paradigm to semistructured, in-person focus groups [17–19]. We chose a constructivist paradigm to understand the lived experience of this unique population. Participants completed a general demographic survey at the start of each focus group. This study was exempted by the UCLA Office of Human Research Protection Program (IRB 23-000885; antibiotic decision making of IM residents), and the primary facilitator (D. A. D.) obtained oral consent from all participants.

### Patient Consent Statement

This study did not include such factors that would require patient consent to be obtained.

### Instrument Development

We designed the demographic survey and focus group script after literature review to optimize content validity [12, 13], using a similar strategy of case recall [4]. The demographic survey and focus group script were read aloud among the study team and piloted with a small representative sample for response process validity. We made minor changes to wording and the ordering of questions for clarity based on feedback from piloting. For the focus group script, we utilized open-ended questions to maximize depth of response, with the intent of creating a participant-driven dialogue exploring how participants approached aspects of antibiotic use generally and recalled patient scenarios specifically, as well as which experiences were most critical to shaping their use of antibiotics during training. We did not make additional changes to the focus group script during data collection. The final versions of the survey and focus group script are available in the supplementary material.

### Study Protocol

Participants completed the demographic survey via Qualtrics on their personal mobile phones or laptops. The facilitator (D. A. D.) was present in person for all 3 sessions, and the senior investigator (T. V.) was present either over Zoom (first and second sessions) or in person (third session). D. A. D. was chosen as the facilitator as a near peer to the participants to minimize any potential perceived hierarchical bias and underwent training with J. M. J., an experienced qualitative researcher, prior to conducting focus groups. D. A. D. read the focus group script in the same manner and order for each session, with occasional prompting to allow a participant to discuss one’s thoughts in more detail or encourage commentary from other participants in response. We audio recorded all focus group sessions and utilized the auto-transcription in Zoom through in-room audio equipment. The facilitator (D. A. D.) reviewed and edited all transcripts for accuracy prior to analysis. We deidentified all transcripts and then uploaded them into Dedoose, a collaborative qualitative analysis software platform for further analysis. We deleted all original recordings after transcript finalization to maximize confidentiality.

### Data Analysis

Two researchers (D. A. D. and T. V.) independently performed thematic analysis of focus group transcripts via iterative coding using a constructivist paradigm [17–22]. The researchers performed open and axial coding, examining data line by line to identify recurring concepts and assign codes, specifically seeking narratives that offered opportunities to broaden, challenge, or disconfirm our evolving themes [18, 19, 21]. Following this review, the researchers established a final coding scheme and reapplied this final scheme to all data independently (D. A. D. and T. V.). They resolved discrepancies through in-depth discussion and negotiated consensus. Overall agreement between the analysts was 85% (codes agreed on/total codes applied). The third focus group did not yield any new codes, insights, or counterexamples. Thus, we determined that we had reached thematic saturation [23, 24]. The researchers converged codes into overarching themes. All in-text quotes are denoted “R1–R3” corresponding to the postgraduate year of the participant trainee.

### Reflexivity

Our study team included an emergency medicine education researcher with advanced training in qualitative research and survey design (J. M. J.), an ID clinician-educator (T. V.) with some experience in qualitative methods, and an IM resident, now ID fellow (D. A. D.). We remained cognizant throughout the study that the characteristics and experiences of the researchers may influence the results. For instance, during data collection and analysis, participants may have felt more comfortable sharing their uncertainty with the facilitator (D. A. D.) as a near peer, as well as the inferences that they perceived to be of most interest. D. A. D.'s transition through ID fellowship training and experiences in antimicrobial stewardship could also have influenced the results and evolution of this article. To address this, we used negative case analysis when we identified outlier data, allowed adjustments of discordant hypotheses as needed, frequently revisited the raw data, and focused our analysis on participants’ actual words rather than implied meaning during coding [18]. To enhance the trustworthiness of our analysis, we used memos to record theoretical and reflective thoughts, and these were subsequently discussed during group meetings [18].

## RESULTS

### Participants

**Figure 1.**
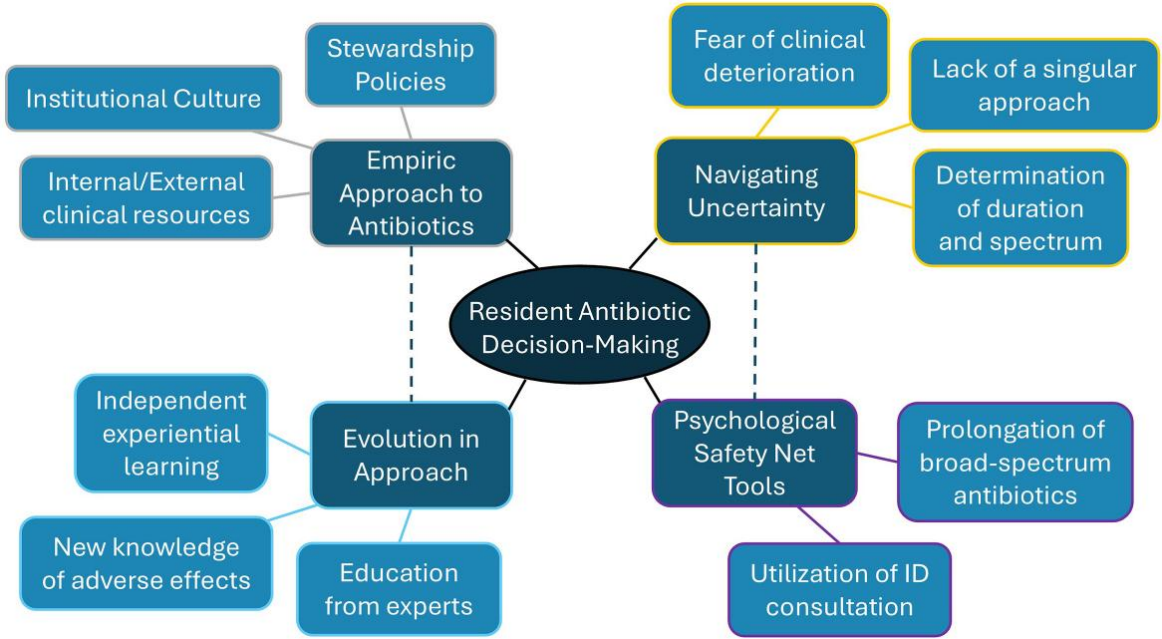
Thought map graphic notes prominent themes and subthemes surrounding resident antibiotic decision making. ID, infectious diseases.

We hosted 3 distinct focus groups with a total of 25 residents. We included all residents who expressed interest and provided verbal consent. Eighteen (72%) completed the demographic survey (Table 1). We identified 4 broad themes pertaining to resident antibiotic decision making: the empiric approach to antibiotics, navigating uncertainty, psychological safety net tools, and the evolution in approach to antibiotic use (Figure 1). These 4 themes comprised an additional 11 subthemes (Supplementary Table 1).

**Table 1.** Participant Demographics.

Demographic	No. (%)
Participants who completed the survey	18 (72)
Age, y, median (range)	29 (26–36)
Training level	
PGY-1	7 (38.9)
PGY-2	8 (44.4)
PGY-3	3 (16.7)
Gender identity	
Nonbinary	1 (5.6)
Female	12 (66.7)
Male	5 (27.8)
Program track	
Categorical	7 (38.9)
Primary care	8 (44.4)
Internal medicine/pediatrics	3 (16.7)
Weeks of night admitting resident	
0	7 (38.9)
1-2	2 (11.1)
3-5	3 (16.7)
6-8	2 (11.1)
≥9	4 (22.2)

Abbreviation: PGY, postgraduate year.

### Empiric Approach to Prescribing

Residents described a clear general approach to prescribing empiric antibiotics, as influenced by the interaction of (1) institutional culture, as defined by generational preferences, transmission from senior to junior trainees, and perceived/experienced risks; (2) stewardship policies, which also dictated hospital formularies and restrictions; and (3) clinical resources. Their empiric approach included the importance of identifying the presence of an infection, the source of infection, the most likely pathogens to be covered, and the presence or absence of critical illness. One intern explained,

I think a lot more about bugs now, like, what bug is it? What bug am I covering? Where is this bug coming from in the human body? Why do they have this infection? (R1)

Participants noted the influence of other patient-specific factors/comorbidities on their empiric decisions: the presence of any immunocompromising conditions, prior microbial data or growth of specific organisms (eg, *Pseudomonas*), and notable allergies. Residents reported deviating from the previous approach and prescribing more broadly and reflexively in patients who were critically ill (frequently referred to as “sick”) and those perceived to be immunocompromised:

We don’t really think about like oh this person has this infection, they have this bug that causes this infection, it’s like no they’re a transplant patient and they’re sick, they’re getting meropenem. And probably vanc too. (R2)

We identified 2 interrelated subthemes influencing the empiric antibiotic approach: the local institutional culture and its interaction with hospital-wide antibiotic stewardship policies. Participants frequently contrasted different prescribing “cultures” (eg, their medical school) with a trend toward modifying one's empiric choices to what is most commonly prescribed in the current training environment. One senior explained,

I’m like much more prone to doing vanc/zosyn than vanc/cefepime, just because that’s just what we do here. (R3)

The differences in stewardship policies and perceived “strictness” of antibiotic restrictions at different training sites modulated empiric choice as well, as one resident brought to attention:

Yeah, at [county hospital], there’s very strict limitations on like using big gun antimicrobials like vanc, and like mero. (R2)

Participants utilized various internal and external resources that aided them in their empiric prescribing, such as institution-specific antibiograms and guidance (through the Firstline app), electronic health record order sets, as well as common online resources. These included UpToDate, Infectious Diseases Society of America guidelines, and Micromedex, among others.

### Navigating Uncertainty

One common challenge faced by residents when prescribing antibiotics was how to best navigate various forms of uncertainty. They described a fear of patients' clinical deterioration directly linked to their use or withdrawal of antibiotics, culminating in a reluctance to de-escalate. This fear of potential deterioration from de-escalation was associated with strong feelings of personal responsibility for negative patient outcomes:

I think it’s more just like the fear of like missing a bacterial infection or not treating something and having someone like decompensate because of that, cause that’s like happened before, too, where we peeled it off too quick and then they ended up like in the ICU. (R2)

Multiple residents acknowledged the cognitive dissonance between prescribing potentially unnecessarily broad antibiotics and the effect that it may have on individual and global resistance patterns. One participant commented,

I think sometimes you end up, leaving them broad, and you just do like a treatment duration. But you’re not like confident that that was actually like the overall best interest of the patient from the standpoint of resistance and things like that. (R3)

Specific populations, namely patients who were immunocompromised and/or young, were particularly challenging to our participants. Exceptionalism and emotional prescribing were common, as the perceived “high risk” status of these patients often led to overcoverage. One intern explained,

She was like pretty young and healthy otherwise other than this, like terrible cancer. So I think there was a lot of like momentum, too, to like, just try to throw everything at her. (R1)

There was notable frustration with the lack of a singular approach to treating infections, frequently exemplified by the variance seen across attending physician practices. Some residents felt unsupported by their attendings in decisions to narrow or de-escalate antibiotics, and they struggled to find consistency in therapeutic reasoning among attendings. These complex decisions often felt “vibes based” rather than being rooted in true data-driven practice, as one resident noted:

It’s like a very individualized decision, based on what the attendings want. And so, there’s like a lack of clear direction or reasoning behind why a lot of these antibiotics are started or decided until you end up not being able to kind of build that knowledge base throughout residency training because the reasoning is inconsistent. (R2)

Determining the appropriate duration and, occasionally, spectrum of antibiotics was a clear gap in understanding, as one senior lamented:

Duration of antibiotics especially are like so vibes based . . . are we gonna treat this for like 5 days or like this is kinda severe so maybe we’ll do 10 or maybe even 14. (R3)

### Psychological Safety Net Tools

When faced with these challenges, residents predominantly utilized 2 strategies to assuage their concerns: ID consultation and prolonging and/or broadening antibiotics. ID consultants were frequently employed as a safety net by “having ID on board.” This was not only for general support but also for validating previous treatment decisions and/or sharing the burden of perceived riskier management choices, such as de-escalation. One junior resident noted,

Even though you get data and we have a thought, we often check on it and confirm with the consultants. We were kinda like, oh we can probably de-escalate, but let’s just double check with ID. (R2)

Even without ID involvement, antibiotics were a catch-all psychological safety net, with a trend toward prolonging or broadening antibiotics in times of uncertainty, as described by one participant:

That aspect of like erring on the side of being broad, and like, I think, honestly, most attendings like preferring and encouraging that as well, to where you’re confident that you covered anything that might be happening. (R2)

### Evolution in Approach

Various aspects of residents' approaches to antibiotic use evolve over residency. Distinct from their previously described role as a safety net, ID consultants as well as ID pharmacists serve as a vital source of education, as one resident noted:

I felt like a lot of my learning comes from our ID consultant. Probably like 70%-80% of the time when it comes to antibiotics. (R2)

Residents benefited immensely by learning the ID framework when working alongside ID consultants, in clinic and on inpatient consults, as mentioned by a junior resident:

I think, like that systemic approach to infections in general was like helpful to build, too, because I feel like that forces you to kind of critically think about it and kind of fine tune your decision-making process accordingly. (R2)

Some residents modeled perceived best practices from other physicians, namely their more senior residents, hospitalists, and other subspecialty attendings. They developed new knowledge of adverse effects of antibiotics from personal experience as well as external resources, such as podcasts. Several commented on the potential for cefepime neurotoxicity, with one intern stating,

Occasionally an interesting case will come up on [a podcast], I think that’s where I got that cefepime study that came out, cause to be honest I would never be like actively searching for ID literature. (R1)

Trainee growth was not limited to just external instruction or learning from experts, however. Senior residents noted how vital it was to their growth to have independent experiential learning opportunities and reflect on the outcomes of their choices, especially on services with less direct supervision, such as overnight rotations.

I think that I feel more confident after doing a lot of night admitting as a second year because I hate calling consultants in the middle of the night, so I just better make the decision myself. (R2)

Key to this evolution was a frameshift from “thinking fast” to “thinking slow” and being more intentional in their prescribing, as one senior resident thoughtfully described:

I try to keep—like hold myself accountable to that of like, not just blindly prescribing antibiotics, and really being mindful about like, what do I actually need to cover? Am I doing this to cover myself? Or am I using this because the patient actually needs this? (R3)

## DISCUSSION

This study illuminates several important insights into trainees' antibiotic decision making. Our focus groups with residents showed their fundamental approach to empiric prescribing and bias toward institutional practices. Residents rely on and sincerely appreciate the expertise of others, namely ID physicians and pharmacists. These data add to the growing literature for this understudied cohort of physicians in the context of antibiotic decision making and suggest pathways for improvement in the future.

Among the trainee-specific literature, our work replicated several factors that guide antibiotic choice: attending influence [9, 12, 25, 26], use of broad-spectrum antibiotics in “sick” patients and/or during times of uncertainty [9, 12, 26], institutional culture [8, 26], lack of a singular approach [8], as well as an awareness of the risk of resistance with broad-spectrum antibiotic use [8, 9, 12, 25]. Contrasting prescribing cultures were infrequently mentioned in these studies, but one of them identified adapting to different institutional cultures as a major barrier to interns' confidence when prescribing antibiotics [8]. We note that culture is in fact dynamic [27] and can change with alterations in workforce (new outside faculty) or the collective experience of a group, as demonstrated here with the described heightened awareness of cefepime-induced neurotoxicity. Our residents may be more acutely aware of these cultural differences as they rotate through 4 distinct hospital systems, in contrast to residents who work in a single health system.

Interestingly, the usefulness of ID consultation as a method to mitigate uncertainty and the vital role of ID consultants as educators described by our participants have not been previously discussed in the literature. This may be supported by data on increasing the volume of ID consults [28] as having ID “on board” becomes more common. Our participants also noted the benefit of learning an “ID framework,” further underscoring the importance of hands-on experience in ID for trainees. Mandatory rotations in ID have been shown to improve stewardship knowledge for IM interns [29], and given that early exposure in training to ID consults is associated with a higher likelihood of eventually applying into ID fellowship, such requirements may continue to pay dividends for the field at large [30]. Additionally, as evidenced by the prior “cultures” that our participants recalled from their medical school experiences, exposure to the ID framework during undergraduate medical education could have an even broader impact given the ubiquity of antibiotic use to all physicians, regardless of specialty. We have described such educational interventions in the preclinical curriculum, using team-based learning sessions to highlight the nuances of choosing between 2 similarly broad antimicrobials (eg, cefepime and piperacillin-tazobactam) [31].

Navigating uncertainty has been a significant focus of study for the last several decades, and an increasingly lower tolerance for clinical uncertainty has perhaps been exacerbated by the COVID-19 pandemic [32–35]. Most medical student assessments, which represent a significant proportion of learning in early undergraduate medical education, are in the form of multiple-choice questions, leaving little room for ambiguity. Yet navigating uncertainty is a hallmark of physicianship, and effective teaching conveys that uncertainty is not to be avoided but acknowledged as a reality of practice that can be identified and managed [36]. Our participants suggested that despite awareness of the global threat of antimicrobial resistance, a low tolerance for uncertainty may result in reaching for broader antibiotics when not needed. Explicitly naming or classifying types of uncertainty is essential in managing it in clinical practice [34, 37], and our residents did not complete this key step. There may be a similar benefit to naming and discussing other pitfalls in mitigating uncertainty, such as exceptionalism (“our hospital has more sick patients than others”) and emotional prescribing (“this patient is young and dying”).

When our results are compared with the literature on more experienced clinicians, many factors previously identified are seen in our study, including preexisting patient characteristics such as allergies [4, 6, 38], comorbid conditions [4, 6, 38], and past infections [4, 6]; current case features such as microbiologic data [4, 6], severity of illness/triaging [4, 6, 38], and diagnostic uncertainty [6, 7, 38]; provider and health care system factors such as use of antibiograms (and other local resources) [4, 6] and institution-specific practices and culture [4, 6]; and treatment principles such as organizational guidelines [6, 7, 38] and the adverse effects of drugs [4, 6, 7]. Much of the granular content that may arise with experience was not explicitly replicated in our study, such as drug characteristics (eg, pharmacokinetics/pharmacodynamics, route, price) [4, 6], individual social factors (eg, anticipated adherence, cost, patient preferences) [4, 6, 38], and parsimony (preference for fewer agents) [4]. Some of these discrepancies may be attributed to differences in experimental design, given our use of focus groups as opposed to individual interviews, but nonetheless highlight potentially intervenable gaps in trainees' developing frameworks.

Residents in our study readily identified at least some portion of the major categories of factors that modulate antibiotic choice as defined by Abdoler et al, which may be considered aspirational given that participants in that study were either ID or hospitalist attendings [4]. Additionally, the residents in our study were starting to focus on direct adverse effects of antibiotics (neurotoxicity), which may be a more tangible negative outcome. Therefore, antibiotics are understood as not necessarily “low risk.” Notably, none of our participants commented on volume overload, gastrointestinal intolerance, or *Clostridium difficile* infection as other untoward direct consequences, the latter a previously identified risk of antibiotic use among trainees [12, 26]. Such direct consequences perhaps carry more weight than the more abstract selection of multidrug resistance. Generating therapy scripts (central to the proposed therapeutic reasoning framework [4]) that would include drug characteristics and patient factors (including adverse effects) requires iteration and an intentional practice that can be reinforced during independent overnight rotations. These rotations are an excellent opportunity to promote growth among trainees by allowing ample opportunities for trial and error to hone their own specific “vibe.”

The concept of a vibe, or gestalt, may not be as nebulous as perceived. Allowing residents to practice navigating the uncertain landscape of antibiotic selection is as imperative as it is for any other example of therapeutic decision making. Perhaps a space for reflective practice following these night-shift rotations could allow residents to solidify their antimicrobial understanding and reasoning, as guided reflection and feedback in this context has been shown to help identify gaps in clinical reasoning [39]. Creating simulations in antimicrobial decision making with trained facilitators, formalizing a management pause in rounds, as previously described [40], and mandating rotations in ID for all IM interns may be similarly actionable steps. However, to be successful, all such interventions and those responsible for them (eg, ID physicians and pharmacists) must be sure to account for the power of emotion and fear and the challenging interprofessional milieu in which residents are positioned during training [15].

There are several limitations to our study. R3s were underrepresented, and our residents' perspectives are unique to their specific training environment and may not be applicable to other institutions. Focus groups are inherently prone to several forms of bias, such as groupthink and the dominance effect [41]. The voluntary nature of our study may have also selected those most willing to share their views. However, the consistent turnout and willingness to participate suggest that such focus groups led by a near peer may be an ideal “low stakes” avenue for further investigation as it leverages the strong interpersonal connections that occur during residency training. Finally, our study included only IM trainees, and there are clear differences in prescribing practices across other specialties that will need to continue to be explored in future studies [5, 42, 43].

In conclusion, resident decision making regarding antibiotic use is challenged by lowered tolerance for uncertainty, which can be mitigated by independent overnight shifts (with opportunities for reflective practice) and by the teaching and guidance provided by ID consultants and ID pharmacists. Investing in specific educational interventions throughout the training timeline (including undergraduate medical education) and during overnight rotations could enhance therapeutic decision making and optimize antibiotic utilization among trainees.

## Supplementary Material

ofag017_Supplementary_Data

## Supplementary Data

Supplementary materials are available at *Open Forum Infectious Diseases* online. Consisting of data provided by the authors to benefit the reader, the posted materials are not copyedited and are the sole responsibility of the authors, so questions or comments should be addressed to the corresponding author.
