# Supplementary material for: Feeling the Vibes: An Investigation Into Resident Antibiotic Prescribing Practices
Source: Open Forum Infect Dis. 2026 Jan 23;13(2):ofag017. doi: 10.1093/ofid/ofag017 (PMC12881688; doi:10.1093/ofid/ofag017)
Supplement: ofag017_Supplementary_Data [file ofag017_supplementary_data.docx]

**Supplementary Material**

Supplementary Table 1. Results of thematic Analysis

| Main theme | Sub-theme | Exemplar quotes |
| --- | --- | --- |
| **Empiric approach**  *Included general questions, as well as patient-specific factors (e.g. age, immunocompromising conditions)* |  | “I think those are like the three things I look at, like how sick are they, what’s the source, and like what have they grown in the past.” [R2]  “…what is their kind of interaction with the healthcare system? Are they frequently hospitalized? Do they have a history of like resistant organisms?” [R3] |
|  | **Institutional culture**  *References to local prescribing practices to the level of individual institutions not otherwise rooted in specific patient scenarios, influenced by values, behaviors, and norms of the trainees and attendings* | “…where I went to Med school was very particular about antibiotics. You didn’t see vanc/zosyn, they were very against it.” [R2]  [When asked about how they learned about prescribing antibiotics] - “I think hearing the words vanc/zosyn a lot…” [R1] |
|  | **Stewardship Policies**  *Comments related to institution-level policies dictating use or restriction of specific antibiotics, often in context of comparisons between training sites* | “I think something that surprised me just like as an intern at [county hospital], a lot of these broader spectrum antibiotics needed ID approval even overnight … but I was surprised by the ones that like when I did [tertiary hospital] night float, that I could broaden to without ID consultation…” [R1] |
|  | **Internal/External clinical resources**  *Explicit mention of a clinical resource used when prescribing* | “…when I do my rotations at [county hospital], we have like our own antimicrobial guide, where they like look at specific culture data, like um, and like microbes that affect our like specific patient population, and like, they have their own like recommendations on like antibiotic choice and duration.” [R1]  “…but you know I feel like as a senior I use Firstline more frequently to - to aid with that decision-making process. You know, antibiogram and Firstline are super helpful” [R3] |
| **Navigating Uncertainty**  *Influenced by additional factors such as exceptionalism, emotional prescribing, and cognitive dissonance* |  | “…if you're on the [Heme/BMT] service, where, like everyone's super immunocompromised because they've all just had bone marrow transplants, like their threshold to start like the craziest antibiotics is super low.” [R3] |
|  | **Fear of clinical deterioration**  *Hesitance or worry about a patient (often “sick”) decompensating, often associated with a reluctance to de-escalate* | “…and you have like all these reasons to de-escalate, just depends on like comfort level, if they look super sick, some people are like, more hesitant to peel off antibiotics.” [R2] |
|  | **Lack of a singular approach**  *Primarily references to ambiguity, differences in attending practice, and lack of specific algorithms* | “I think that it seems like [antibiotic prescribing] should be super algorithmic. But there's actually so much choice that you have with different anti- and there's so many different ones that cover different, you know, cover overlap in what they cover. And so it's hard like you said, to gain like a logical thought process on how to approach it, because every attending uses some variation, because there's so many options.” [R3] |
|  | **Determination of duration and spectrum**  *Comments related to difficulty determining the appropriate duration of therapy and/or spectrum* | “Even the guidelines are like, up to 7, or 3 to 5, and I just feel like, 7 to 10, even that, 10-14 is my least favorite cause I feel like there’s a big difference between 10 and like I don’t know, two weeks. I don’t know, I think that’s challenging.” [R2]  “Then we end up narrowing either way. But it's like - I'm still figuring out like, why do we broaden so aggressively? And then just narrow two days later, with nothing really changed, you know? I don't know.” [R1] |
| **Psychological safety net tools**  *Describing strategies employed to mitigate uncertainty* |  | “I would much prefer to err on the side of maybe like making sure that we have pseudomonal coverage on board, and then peeling it off with, once we get more data, or the blessing of our ID team, versus the other way around.” [Chief Resident] |
|  | **Prolongation of Broad-spectrum antibiotics**  *Mention of extending or prolonging antibiotics as a result of uncertainty* | “Yeah, and I feel like as an early trainee, like I err on being overly cautious. So I will just treat - you know, do a longer course.” [R1] |
|  | **Utilization of ID consultation**  *References to the involvement of ID consult team, often for reassurance, or a proverbial “blessing” in challenging or unclear cases* | “And then like after a few days – no we like talked with ID again because we were like we don't know what to do.” [R2]  “It's really helpful to have infectious disease on board for them to kind of help us look for that nidus of infection, when we're kind of like - we're not totally sure, we're kind of going based off of previous imaging. And it's still a little bit fuzzy and unclear.” [R1] |
| **Evolution in Approach**  *Comments related to how any aspect of antibiotic prescribing has changed over the course of training, and the factors that influenced this growth* |  | “So I think with time and experience you'll get like even more confident with, like your stewardship, and like peeling back with like just like knowledge of like, how patients tend to trend and also becoming okay with like making mistakes every now and then.” [R3]  “I think, like as you progress, you're like, okay, like, think about the bug, the source. Like all that stuff like I think I forget what the term is called but there’s like a quick way of thinking, and there's like a second way of thinking where you're like, kind of really like actually like thinking about the - the issue. So um, but I think that's gotten easier with time, whereas I think the cognitive load, as a second year, is not as high as like an intern.” [R2] |
|  | **Education from experts**  *References to the importance of received education from ID physicians and pharmacists, as well as learning a specific approach to prescribing* | “I ended up actually doing an ID consult rotation this year, because there’s just like, not enough teaching here on how we came to these decisions and how we’re making our approach to antibiotics, and what the right way is […] and that was very helpful, but outside of that, you don’t get a lot of teaching in regards to antibiotics.” [R2]  “I feel like it’s one of my weaker areas actually, like infectious disease, I feel like we defer to ID a lot, like I’ve been asking ID can you explain to me your thought process with doing this?” [R1]  “ID pharmacy specifically is like, is amazing.” [R2] |
|  | **Independent experiential learning**  *Comments from seniors (R2-R3’s) on their development as prescribers predominantly in the context of independent overnight rotations that lack direct supervision* | “And it kind of reminds me when I did my first NAR rotation, somebody's advice was like, look [name], you're just gonna have to make two decisions overnight: are you gonna diurese or give them fluids? Broad spectrum antibiotics or not? ... And I'll like, admit, I just started everyone on broad spectrum antibiotics if I thought they were infected. Um, but hopefully, knock on wood, I've gotten a little bit more, um I don't know, a little - a little better at that, or like a little bit more nuanced in that decision-making.” [R3]  “I think, like overnight, something that I’ve found that is difficult like you, you should feel empowered to like, not continue what the ED gave, like you don't have to continue what the ED gives.” [R2]  “I think as you do [overnight admitting rotation] more, you feel more and more comfortable, diverting from the plan that you've been handed off, and really thinking for yourself.” [R3] |
|  | **New knowledge of adverse effects**  *Comments related to a new appreciation for side effects of antibiotic use causing patient harm* | “But I've seen so much like neurotoxicity with cefepime that like I, I'm like a little bit more kind of scared to use that nowadays.” [R3]  “I think that, understanding that antibiotics have side effects, uh like vanc/cefepime I’ve had a couple end up on dialysis … and like, I don’t know, what we were treating at the time, but just like acknowledging that like, there's always a risk/benefit with any combination … especially with broader spectrum, but any antibiotic...” [R2] |

Demographic Pre-survey

Administered through Qualtrics.

Thank you for your interest in our medical education project. Please answer the brief demographic questions below.

Unique Identifier creation. Please enter the last letter of your first name, followed by the first two letters of your last name, followed by your birth month (MM format).

1. What is your age (in numbers)?

2. What year of residency are you currently in?

a. PGY-1

b. PGY-2

c. PGY-3

d. PGY-4

3. What gender (if any) do you identify as?

a. Prefer not to say

b. Non-binary / third gender

c. Female

d. Male

4. Which residency program are you a part of?

a. Categorical

b. Primary Care Track

c. Med/Peds

d. Prelim/Affiliated program (please enter)

e. Other

5. Approximately how many weeks of [hospital names] NAR (Night admitting resident) have you completed?

a. 0

b. 1-2

c. 3-5

d. 6-8

e. >9

Focus Group Script

-Tell me about your typical approach to prescribing antibiotics.

-How have you learned about how to prescribe antibiotics?

-Has anything surprised you about the use of antibiotics in the hospital?

-Has anything surprised you about the way you utilize antibiotics?

-In what ways has your approach to antibiotic use evolved during your residency training?

-What kinds of experiences during residency were particularly impactful on your antibiotic prescribing practices?

-Think back on a recent patient you cared for that had a very clear infection requiring treatment during their hospitalization. How did you choose antibiotics for this patient? What factors influenced this decision?

-Reflect on a case where antibiotics, or a specific antibiotic, were stopped on a patient that you cared for. What factors influenced your decision to withdraw these treatments?

o If there are no obvious cases that come to mind, instead consider a case where antibiotics were de-escalated.

-Are there specific clinical resources or decision-making tools that you use when managing a patient presenting with an infection? If so, please describe them.

- Can you describe any specific situations where you typically use these resources? Can you describe how frequently you use them?

-Think back to the last time you prescribed antibiotics in the inpatient setting. What (if any) resources did you use for selecting the antibiotics and determining appropriate dosages and frequency?

- What challenges, if any, have you encountered when ordering antibiotics? How have you navigated those? In what ways can the antibiotic prescribing process be improved?

-How confident do you feel in your antibiotic prescribing decisions? Is there anything that would help improve your confidence in these decisions?

-Is there anything else you would like to share regarding antibiotic use?

Thank you all again for your participation.
